# Chronic *Trichuris trichiura* Infection Presenting as Ileocecal Valve Swelling Mimicking Malignancy

**DOI:** 10.5402/2011/105178

**Published:** 2010-10-31

**Authors:** Sharifah Emilia Tuan Sharif, Ch'ng Ewe Seng, Nazri Mustaffa, Nurul Azira Mohd Shah, Zeehaida Mohamed

**Affiliations:** ^1^Department of Pathology, School of Medical Sciences, Universiti Sains Malaysia, Kubang Kerian, Kelantan 16150, Malaysia; ^2^Department of Medicine, School of Medical Sciences, Universiti Sains Malaysia, Kubang Kerian, Kelantan 16150, Malaysia; ^3^Department of Medical Microbiology and Parasitology, School of Medical Sciences, Universiti Sains Malaysia, Kubang Kerian, Kelantan 16150, Malaysia

## Abstract

A 46-year-old man presented with a history of passing bright red blood per rectum over the last one month. He also had on and off diarrhea with visible mucus in the stool for two months' duration. Further history was unremarkable, and physical examination revealed hemorrhoids which were subsequently banded. A colonoscopy was arranged in view of the prolonged diarrhea whereby an edematous and swollen ileocecal valve was seen. This was shown to be due to *Trichuris trichiura* infection, confirmed on histopathological examination of biopsies taken from the site. The patient was started on oral albendazole treatment and has been asymptomatic on latest followup. This case illustrates an accidental finding of *T. trichuria* infection on colonoscopic examination, which was done to investigate the patient's prolonged diarrhea.

## 1. Introduction

Prolonged diarrhea may pose a diagnostic problem in clinical setting, especially in differentiating between infective, inflammatory, and malignant causes. In tropical countries, the infectious causes of diarrhea may be high in the list of possibilities. However, causes by malignancy would also be possible when patients presented with passing of blood per rectum. Among the infectious causes in adult patients, bacterial and viral pathogens are mostly commonly implicated and looked for during the diagnosis. Many people had forgotten about the possible parasitic etiology for diarrheal disease; *Giardia lamblia, Entamoeba histolytica,* and *Trichuris trichiura* are among the examples. *T. trichuria* infections may present symptoms in heavy parasitic infection while others may be asymptomatic. The symptoms include anaemia, diarrhea, and intestinal bleeding [1]. The diagnosis is made by identifying *T. trichiura *eggs in stool specimens.

Several reports have described the detection of *T. trichiura *during colonoscopy; recently, trichuriasis was diagnosed incidentally by detection of adult worms on colonoscopy of 4 patients. Colonoscopy was performed for evaluation of their nonspecific gastrointestinal symptoms, such as abdominal pain, diarrhea, and anemia. Moreover, stool examinations were all negative for both eggs and parasites in those cases [2]. The purpose of this case is to highlight the accidental finding of *T. trichuria* infection on colonoscopic examination which is the first encounter in our setting.

## 2. Case History

**Figure 1:**
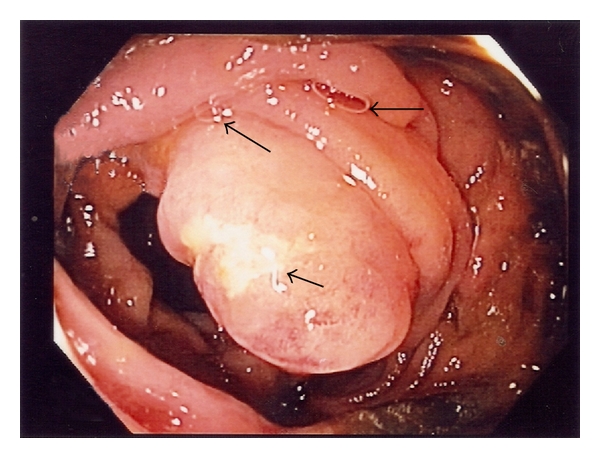
A swollen ileocecal valve with superficial ulceration. A few thread-like nematodes embedding the intestinal mucosa were seen (arrow).

A 46-year-old man complained of two episodes of bright red rectal bleeding during the past month. He also had diarrhea intermittently for the past two months associated with visible mucus in the stool. There was no significant previous medical illness, and physical examination was also unremarkable. Proctoscopic examination showed two internal hemorrhoids which were banded. Due to the history of prolonged diarrhea, colonoscopy was arranged for him. This revealed a swollen ileocecal valve associated with superficial ulceration (Figure 1). Biopsies were taken to rule out possible malignancy. The current diagnosis was internal haemorrhoids with possibility of colonic malignancy. A simple stool for microscopic examination was not ordered for him since the clinical diagnosis pointed towards malignancy.

**Figure 2:**
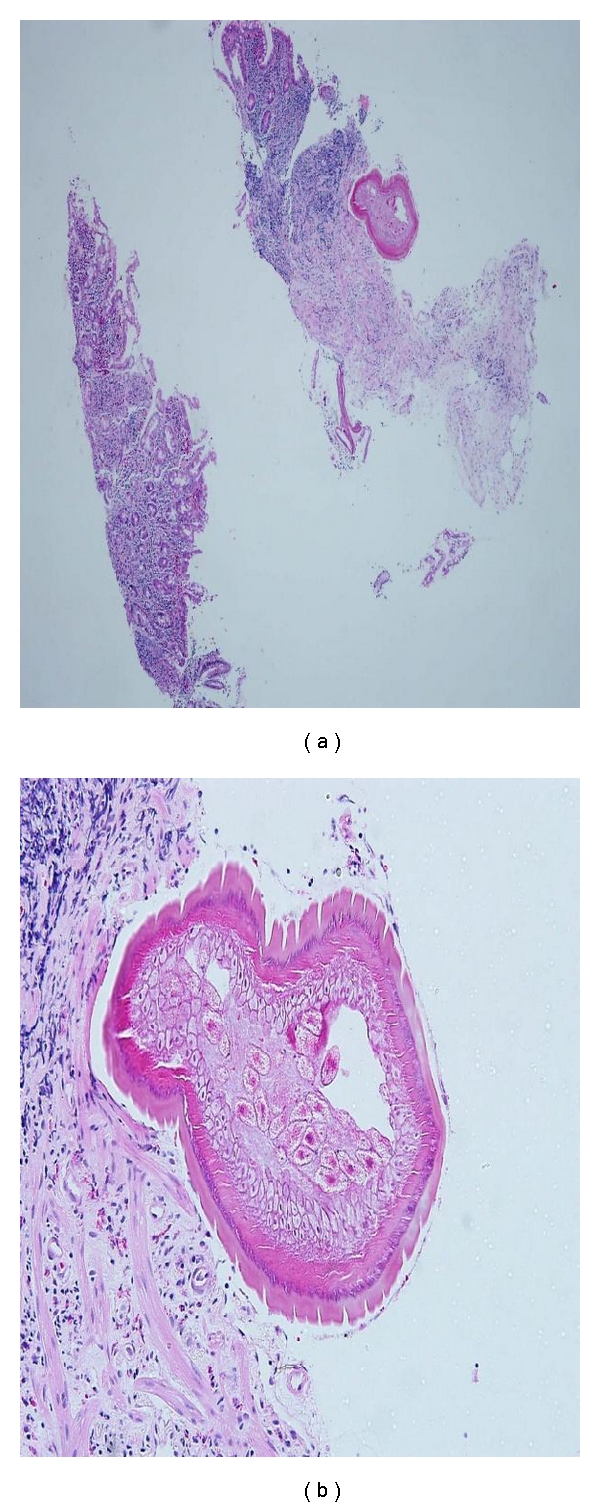
(a) 40x H&E ileocecal valve showed moderate lymphocytic cell infiltrates in the mucosa together with cross sections of an adult female nematode. (b) 200x H&E ileocecal valve showed moderate lymphocytic cell infiltrates in the mucosa together with cross sections of an adult female nematode.

**Figure 3:**
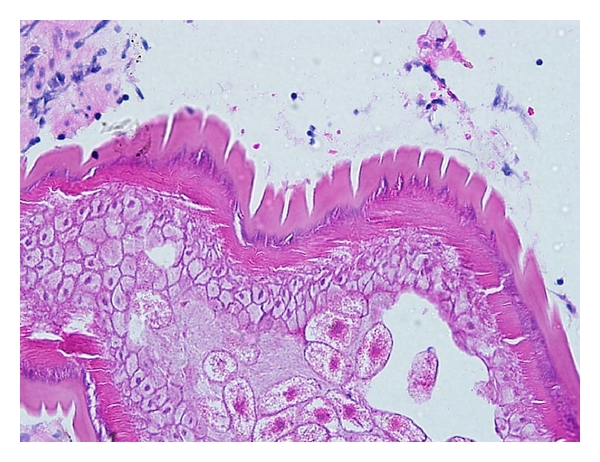
400x H&E Cross section of the worm body wall displayed cuticle with prominent annulations, hypodermis with hypodermal nuclei, contractile portion muscle, and sarcoplasmic portion of muscle with nuclei.

**Figure 4:**
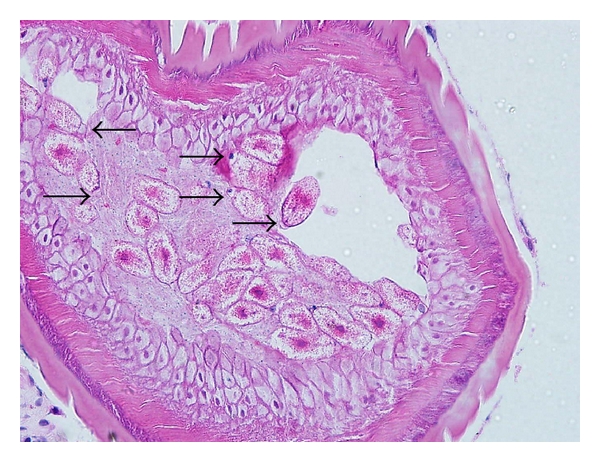
400x H&E Multiple ova with terminal plugs observed within the body (arrow).

**Figure 5:**
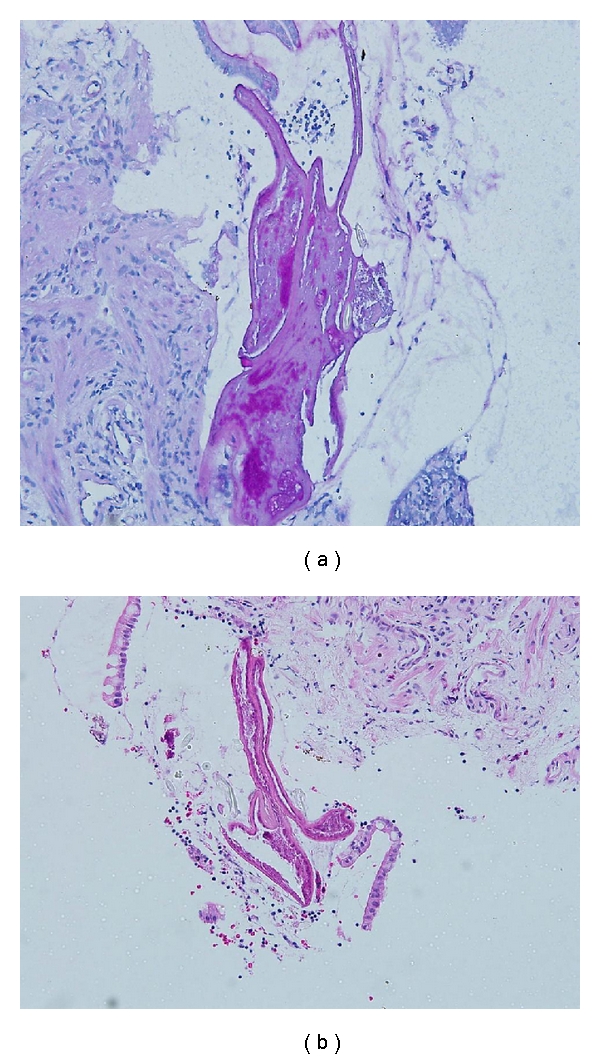
(a) 200x H&E. (b) PAS. Cross section of caudal end showing two caudal papillae.

Histopathological examination of the biopsied tissue from the ileocecal valve showed moderate lymphocytic cell infiltrates in the mucosa together with cross sections of an adult female nematode (Figure 2). The cross section of the worm body wall displayed cuticle with prominent annulations, hypodermis with hypodermal nuclei, contractile portion of muscle, and sarcoplasmic portion of muscle with nuclei (Figure 3). Multiple ova were observed within the body, and these ova appeared to have terminal plugs (Figure 4). The cross section of the caudal end revealed two caudal papillae (Figures 5(a) and 5(b)). These findings were consistent with an adult female *Trichuris trichiura. *He was treated with oral albendazole 200 mg daily for three days and remained asymptomatic on followup several months later.

## 3. Discussion

*Trichuris trichiura* is one of the commonest soil transmitted helminthes prevalent in tropical countries. Infection is acquired by ingestion of embryonated eggs. The egg shell is digested in the small intestine where the larva lodges temporarily. After a period of growth, larvae passed to the cecal area by permanent attachment of anterior ends in the mucosa [3]. The natural life cycle of this helminthes explained the ileocecal valve involvement in this case report. Most infected patients present with diarrhea while others are asymptomatic [4]. It is known to cause chronic infection and in severe forms may be complicated with rectal prolapse, especially in children [5]. 

Detection of this worm during colonoscopy occurs accidentally in most reported cases [2, 6]. In South Korea, 13 patients were diagnosed to have *T. trichiura* infection during colonoscopic examinations in an 11-year review. Four patients were asymptomatic while others presented with diarrhea, abdominal pain or discomfort, and tenesmus. Only two patients had demonstrable ova in stool samples, reflecting the fact that ova are not found on stool examination in the majority of patients [2]. Generally, stool microscopy for ova detection depends on worm burden and has a low sensitivity especially in light infection. Due to this, it is recommended to use the concentration method to increase the yield of detection [7].

Endoscopic findings regarding *T. trichiura* infection are scarce. Colonoscopy may only show normal appearing mucosa with no other abnormalities and numerous thin, whitish, coiled worms [6]. With careful inspection, however, the thin head portion of the worm can be seen embedded in the mucosa while the thicker portion may be visible within the lumen which suggests a diagnosis of *T. trichiura* infection [2]. This might be missed by inexperienced persons performing the colonoscopy procedure.

Colonoscopy has been suggested to be carried out in cases with chronic diarrhea and anemia. This allows direct visualization of nonspecific mucosal inflammation, as well as adult worms attached to mucosa especially within the cecum [6]. It is also performed for evaluation of nonspecific gastrointestinal symptoms, such as abdominal pain, diarrhea, and anemia. From previous case reports, colonoscopic procedures were suggested to be a useful diagnostic tool, particularly when patients are infected with relatively few male worms and are negative for infection on stool microscopic examination [2].

Our patient is the first case to be reported from this hospital on an accidental finding of *T. trichiura* infection during colonoscopy, which was done for investigation of suspected colonic malignancy. The unexpected association of *T. trichiura* in this patient gave a substantial finding of educational value to the doctors dealing in such cases. *T. trichiura* infection has been reported to manifest as colonic hyperemia, edematous mucosa, and multiple erosions as a result of inflammatory changes [8]. A majority of those who are colonized by a small number of worms are asymptomatic. However, persons with a greater worm burden may present with anemia, diarrhea, abdominal pain, weight loss, malnutrition, appendicitis, colonic obstruction, perforation, or intestinal bleeding [1]. In addition, other clues mentioned in the literature such as localized eosinophilic infiltrations without definite eosinophilia may be helpful for diagnosis [2]. However, eosinophilic infiltration was not seen in our patient.

The standard regimen for treating whipworm infection is a three-day course of either oral mebendazole 100 mg twice daily or oral albendazole 400 mg once daily [2]. The complete cure rate of the mebendazole regimen may range from 60% to 100% [4, 9]. Treatment with single dose of either mebendazole or albendazole is not recommended as these were found to have an unsatisfactory cure rate of less than 40%. In such situations, repeated treatment has been suggested three weeks after initial therapy [6]. 

Oral mebendazole 100 mg twice a day for 3 days duration has been proven to be effective (approximately 70% cure rate) and is safe in the treatment of *T. trichiura* [10]. Single-dose albendazole regimens were associated with low success rates. Cure rates for infection with *T. trichiura *following treatment with single-dose oral albendazole and mebendazole were 28% and 36%, respectively [11]. However, a better cure rate of up to 80% is achieved when the duration of treatment is extended for up to 3 days [12–14]. In some case reports though, patients may need a longer duration of treatment (5–7 days) to improve, in patients with severe infection [15]. 

The clinical manifestation of chronic *T. trichiura* infection may mimic other diseases that could lead to delay in initiating treatment. In view of low awareness levels among doctors who are directly in contact with patients, ongoing education regarding *T. trichiura* infection, for example, parasite morphology for those in related fields (surgeons, gastroenterologists, and pathologists), would help to identify cases and thus prevent further complications. As a suggestion, colonoscopy of cases with high index of suspicion has been shown to be the best alternative method other than routine stool microscopy for the diagnosis of *T. trichiura* infection, especially among patients with mild infection since in these patients the stool microscopy will be negative [2, 16].

## 4. Summary

Clinical manifestations of *T. trichiura *infection may vary widely. Mild cases will be underdiagnosed and follow a chronic course of the infection. The presence of other concurrent diseases among patients with chronic trichuriasis may give misleading clinical features of a combined disease. Thus, this clinical scenario may subject the patients to various invasive and expensive investigations while the cheapest test will give result and confirm the diagnosis. Certain cases with mild infection will be an exception. In this case, a combined effort of clinical suspicion, endoscopic findings, and histopathological identification of worm in tissue section may improve the diagnosis and, hence, facilitate the correct treatment of the infection.
